# The value of a short practical training course for newly qualified therapists working with children with cerebral palsy in South Africa

**DOI:** 10.4102/ajod.v9i0.610

**Published:** 2020-04-21

**Authors:** Takondwa C. Bakuwa, Sonti Pilusa, Gillian Saloojee

**Affiliations:** 1 Department of Physiotherapy, College of Medicine, University of Malawi, Blantyre, Malawi; 2 Department of Physiotherapy, University of the Witwatersrand, Johannesburg, South Africa; 3 Malamulele Onward NPC, Johannesburg, South Africa

**Keywords:** continuing professional development, newly qualified therapists, cerebral palsy management, short practical courses, South Africa

## Abstract

**Background:**

Cerebral palsy (CP) is the most common and most complex disabling disorder in children. Newly qualified therapists are expected to manage CP despite feeling inexperienced and inadequately prepared. Short postgraduate practical training courses could potentially help bridge this readiness gap. However, the value of these short courses in addressing the knowledge and experience gap is unknown.

**Objectives:**

To establish the value of a short practical training course on the self-perceived readiness of newly qualified South African trained therapists to work with children with CP.

**Method:**

Secondary analysis of records on therapists’ immediate evaluation of a short practical training course on CP management was completed. The analysis included records from 11 courses collected over a 2-years period (2015–2017). Paired *t*-tests were used to determine the change in knowledge in the quantitative questionnaire. Qualitative data were analysed inductively to determine themes.

**Results:**

The majority of therapists had their expectations met by the course. Therapists’ self-perceived level of knowledge about various aspects of CP after the course changed significantly. Therapists appreciated the adult teaching and learning methods, conducive learning environment, the relevant and organised content and holistic approach of the course. They demonstrated readiness to adopt positive attitudes, perceptions and practice following the course.

**Conclusion:**

A short practical postgraduate training course in CP is valuable in addressing the self-perceived lack of readiness amongst therapists with little experience in this area. It is capable of improving the knowledge and changing attitudes, perceptions and practice intentions positively, and thereby potentially improving the quality of service offered to children with CP.

## Background

Newly qualified therapists in South Africa are required to complete 1 year of mandatory community service after their 4-year undergraduate training as a means of increasing human resource in underserved areas (Naidoo, Van Wyk & Waggie 2017; Singh, Booth & Cholo 2015). However, there is evidence that newly qualified rehabilitation therapists in South Africa, including speech therapists, occupational therapists and physiotherapists, do not feel ready to work in primary healthcare settings. Studies have highlighted the inability of undergraduate training to adequately foster resilience, reflective practice and contextualised learning (Mostert-Wentzel, Frantz & Van Rooijen 2013a; Ramklass 2009a). Inadequate nurturing and lack of support provided to newly qualified therapists have also been reported (Naidoo et al. 2017; Van Stormbroek & Buchanan 2016). Given the fact that newly qualified staff members feel unprepared for work in primary health settings and in resource-limited environments, a need for continual professional training after graduation exists.

Recommendations have been made regarding continuing professional development (CPD) for newly qualified therapists already practising in South Africa (Singh et al. 2015). Suggestions include the formation of virtual communities of practice, mentorship programmes and postgraduate focused short training courses (Mostert-Wentzel, Frantz & Van Rooijen 2013b; Naidoo et al. 2017; Van Stormbroek & Buchanan 2016). Focused training courses in the form of CPD short courses can equip rehabilitation therapists with practical evidence-based techniques and communication skills to foster therapeutic relationships and experiences of working in a local community setting (Grace et al. 2017; Gunn & Goding 2009; Singh et al. 2015).

Postgraduate training courses employing adult learning principles that are largely practice based have been found to be ideal for enhancing learning that is relevant to ‘real-life’ situations, and therefore improving competence (Grace et al. 2017; Singh et al. 2015). The adult learner prefers an environment that is non-threatening to self-expression, which allows mutual feedback, experiential learning and active engagement in practical learning (Chipchase, Johnstone & Long 2012). In a South African study, dieticians, occupational therapists and physiotherapists reported a preference for formal practice-based CPD forums where they could learn from experts (Van Vuuren & Nel 2013). They perceived that observing experts demonstrating practical skills would enhance skills acquisition (Van Vuuren & Nel 2013).

Cerebral palsy (CP) is one of the most complex health conditions occurring in childhood as it includes a variety of associated impairments that limit functional activity and participation in a child’s life (Brew et al. 2018). Cerebral palsy remains a challenge in Africa as children with CP tend to present with more severe degrees of disability compared to other cohorts worldwide (Bearden et al. 2016; Cooper 2015). Being a lifelong condition, the needs of a child with CP will vary with age, family circumstance and the availability of resources in the community setting. Cerebral palsy is therefore an area that newly qualified therapists are likely to find difficult to manage, particularly in a low-resource setting where hospitals have a high caseload of children with CP (Adan 2016).

There is limited evidence on the extent to which CP is regarded an area of difficulty for occupational and physiotherapists. However, in a few studies, newly qualified speech therapists have reported difficulties with managing CP, especially in the area of dysphagia (Singh et al. 2015; Wranz 2011). In these studies, speech therapists perceived themselves as having the required knowledge but lacking in confidence with skills to deliver services at community levels (Singh et al. 2015; Wranz 2011). Both studies highlighted the need to strengthen undergraduate curriculum so that it would provide more opportunities for the acquisition of practical skills. The studies also highlighted the need for continued learning opportunities for the newly qualified therapists.

To bridge the gap of CPD in the area of CP, Malamulele Onward, a South African non-profit organisation (NPO number: 2006/032287/08) based in Johannesburg, has been offering practical training courses on CP for newly qualified therapists since 2014. The organisation’s aim is to offer innovative solutions to improve the quality of life of children with CP and their families living in rural resource-constrained settings. It is the first organisation to champion parent-led CP programmes in South Africa (Burton 2015), which has contributed to reduction of burden of care and improvement of quality of life amongst parents and caregivers (Adan 2016; Burton 2015). Their influence and contribution to CP care extends to most parts of South Africa and neighbouring countries like Lesotho and Rwanda (Burton 2015). Whilst the value of its work with parents and families of children with CP has been evaluated in various ways, the value of the short training courses for therapists working in the rural and low-resourced areas has not been formally evaluated.

This study was conducted as the first step towards evaluating the value of the short practical training courses for therapists offered by Malamulele Onward. Evaluating the value and impact of formal CPD short courses is imperative for short course programme improvement (Campbell, Taylor & Douglas 2017). The authors realise that course evaluation is a multi-stage process that can be done at different levels, namely, the effect on the participants’ self-efficacy, the effect on professional behaviour and the effect on healthcare outcomes (Sinclair et al. 2016). Moreover, the evaluation can be divided into four logical steps that trace the effects of training in a successive order or interlined manner (Campbell et al. 2017; Coldwell & Simkins 2011). The first level identifies participants’ reaction to the training course. This includes satisfaction with the course delivery and also deliverables. The second level assesses participants’ learning in terms of acquisition of knowledge and skills, whilst the third level assesses transferability of learning in terms of change in behaviour of practice. The fourth level assesses desired results in terms of the overlying impact of changes in practice for the institution or organisation (Campbell et al. 2017; Coldwell & Simkins 2011). The scope of the evaluation in this study describes the first stage of evaluation that captures participant reactions, including self-perceived change in knowledge, attitude and clinical practice intention.

Hence, the aim of this study was to establish the value of a short practical training course in the management of CP for newly qualified therapists. This will contribute to the body of knowledge in postgraduate CPD course evaluation considering that there is a paucity of data on evaluation of courses by therapists in South Africa. Specific objectives of the study included the following: to establish whether the expectations of the therapists who attended the CP practical training course were met; to determine the change in self-perceived knowledge regarding CP amongst the therapists as a result of completing the course; to describe the self-perceived change in attitude, perception and intention to clinical practice regarding CP amongst the therapists as a result of completing the course; and to describe elements perceived as essential components of a short course on CP by the therapists.

## Methods and analysis

### Study design

This study was a secondary analysis of course evaluation data collected during 11 courses offered by Malamulele Onward between 2015 and 2017.

### Course description and structure

The short practical course for rehabilitation therapists comprised 6 days of intensive teaching, practical treatment, discussions and reflection sessions. The objectives of the course included equipping therapists with practical understanding of the different types of CP, classification of CP, treatment and management ideas for the different types of CP, functional goal setting according to the type of CP, incorporation of play, communication and functional visual training into the child’s daily routines, basics on feeding techniques (safe eating and drinking), implementing a 24-h postural management programme, group rehabilitation, making basic equipment for children with CP and working effectively and respectfully with parents and caregivers. The course was largely practice-based and participatory. It was administered by an interprofessional team of experienced speech therapists, occupational therapists and physiotherapists. Actual children with CP and their caregivers were involved in all courses to reinforce the practical sessions. The methods of course delivery, approach to learning, the theory content, skills and experience of facilitators were consistent over the period included in this study. The course was open to all therapists (physiotherapists, occupational therapists and speech therapists) working with children with CP and was accredited 30 CPD points. The majority of the therapists attending the course were newly qualified therapists.

Participating therapists completed a general information form that captured details of the background of participants, including their professions and workplace (Appendix 1). In addition, the form also captured information on expectations of the therapists from the course. Participants also answered a questionnaire on knowledge intended to determine their perceptions of their baseline level of knowledge regarding the different aspects of CP addressed in the course (Appendix 2). At the end of the course, the questionnaire on knowledge was administered again. Participants were asked to rate their knowledge again to determine their post-training level of knowledge. At this point, the therapists were asked to rate their knowledge before the course again so as to actually capture a true reflection of their perceived knowledge before the course ‘now that they have been on the course’. Against this, they were asked to rate what they perceived as their new level of knowledge following the course. Participants also completed a course evaluation form that included sections on overall satisfaction with the course, feedback on course delivery and learning (Appendix 3).

All course participants were informed that the forms would be used to formally assess the courses at a later stage. The participants were given the option to indicate if they did not want their records to be included in future research. In this way, facilitators of the course obtained verbal informed consent for use of the records for research purposes.

### Study participants

The study included all records of therapists who attended the Malamulele Onward 6-day training courses between April 2015 and May 2017.

**Inclusion criteria**: Records of speech therapists, physiotherapists and occupational therapists with equal to or less than 4 years of working experience since obtaining their undergraduate qualification.

**Exclusion criteria:** Records of mid-level workers or non-therapists (e.g. dieticians) who attended the course and records of therapists with greater than 4 years of experience since obtaining their undergraduate qualification.

### Data collection tools

Data were collected by using three tools:

- The General Information Form (Appendix 1) which was designed to capture the demographic data of the therapists, including their professional background and practice experience. It also sought information on the expectations of the therapists from the course.
- The Knowledge Questionnaire (Appendix 2) which was a 15-item closed-ended questionnaire. Therapists were asked to rate their own perceptions of their level of knowledge regarding the 15 different aspects of CP on a scale of 1 to 10. The same questionnaire was administered twice: before and after the course.
- The Evaluation Form (Appendix 3) which comprised both closed- and open-ended questions designed to seek feedback on the delivery of the course, overall satisfaction with the course and readiness to change clinical practice behaviours. The last part of this form was evaluation in terms of the ‘way forward’. There was a special prompt that was given to help participants identify what they would do differently following the course. The special prompt was an open-ended prompt: ‘Before I used to…now I will…’ (see Appendix 3).

### Validity and reliability of the tools

The tools used to collect evaluation data were developed by the Malamulele Onward therapy team comprising a physiotherapist, occupational therapist and speech therapist in 2014. The tools were based on their clinical experience as well as the objectives of the course. The aim was to develop evaluation tools that could adequately assess both the theory and application to clinical practice and service delivery as stipulated by the family-centred approach (FCA) (McDowell, Duffy & Parkes 2015). This would also constitute valuable feedback for the course facilitators in terms of areas needing improvement as well as a greater understanding of the participants’ needs and their current practice. The original Knowledge Questionnaire contained 17 items. The General Information Form had 10 sections, whilst the Evaluation Form had five sections. The tools were piloted on one of the first courses in 2014 for face validity and clarity. Areas of apparent lack of clarity, redundancy or incompleteness were identified. The tools were revised by the same team in 2014. The tools were piloted again in 2015 in the subsequent course.

The final version of the Knowledge Questionnaire therefore contained 15 items. The General Information Form maintained the 10 sections. A section that specifically prompted participants to contrast former tendencies and new things they were planning to adopt after the course was added to the Evaluation Form.

### Data analysis methods

A mixed-methods approach was used to analyse the data. Quantitative data regarding the profile of participants were analysed using frequencies and means, whilst change in the self-perceived level of knowledge was determined using paired *t*-tests. All quantitative analyses were performed using STATA software package version 14. Qualitative data analysis was based on Kirkpatrick’s level model framework for course evaluation as described by Campbell et al. (2017). Particularly, the analysis focused on Kirkpatrick’s level 1 of course evaluation, which describes the reaction of participants to a course delivered to them (Campbell et al. 2017). The qualitative data were extracted from the forms and collated into three Microsoft Word documents with predetermined categories, namely, expectations, aspects identified as useful and intention to change. Categorical content analysis was completed for each group of text, which involved coding of data and organising codes with similar foci into categories and subcategories (Gondim & Bendassolli 2014). MAXQDA software package was used to manage and sort the codes.

Dependability and confirmability were the two elements of trustworthiness which were particularly important for this study as it was a secondary analysis (Anney 2014; Cope 2014). To ensure dependability, the data underwent a code-recode process, whereby all three of the researchers involved analysed selected sections of the data independently to check for agreement in the codes. In the case of disagreement in the codes, data were re-analysed until agreement was achieved. To demonstrate that the ultimate interpretations truly originated from the participants’ own words and not the researcher’s own biases (confirmability), the researcher reviewed all 163 records available with the aid of the MAXQDA software analysis package. All responses were considered and inductively built from singular units to codes and into overarching categories.

### Ethical considerations

Ethical clearance to conduct the study was obtained from the Ethics and Research Committee of University of the Witwatersrand (Ethical Clearance number: M170833).

## Results

### Study records

Eleven courses were completed during the 2-years study period (i.e. April 2015 to May 2017), yielding a total of 223 participant records. Sixty records were excluded as they were records of participants who had more than 4 years of work experience and/or records of participants who were non-therapists. Therefore, 163 records were included in the study. However, a minimum of 129 records were used to determine the change in level of knowledge. This is because some records had missing scores on the questionnaire on knowledge and hence paired *t*-tests could not be performed for those specific records. A power analysis computed in Stata/IC version 14, using a sample of 129 records, provided a 100% power to detect an effect size (standardised mean difference), assuming 5% significance level.

### Profile of participants

The majority of course participants had less than 2 years of work experience and were from all of the eight South African training universities. The participants also worked in all the provinces except Limpopo. More than half (*n* = 91; 56%) of the participants were occupational therapists, as summarised in Table 1.

**TABLE 1:** Profile of participants who attended the course.

Variable	Number (*n*)	Percentage
**Discipline to which participants belonged (*n* = 163)**		
Occupational therapy	91	56
Physiotherapy	47	29
Speech therapy	25	15
**Years of working experience since undergraduate qualification (*n* = 163)**		
< 1 year	111	68
2 years	31	19
3 years	10	6
4 years	11	7
**University where participants were trained (*n* = 163)**		
University of Cape Town	32	20
University of KwaZulu-Natal	32	20
University of Stellenbosch	31	19
University of the Free State	22	13
University of Pretoria	18	11
University of the Western Cape	15	9
University of the Witwatersrand	8	5
Sefako Makgatho University	5	3
**Province where participants were working (*n* = 163)**		
KwaZulu-Natal	58	36
Eastern Cape	46	28
Mpumalanga	18	11
Gauteng	15	9
Free State	9	6
Northern Cape	7	4
North West	5	3
Western Cape	3	2
Not working yet	2	1
Limpopo	0	0

### Participants’ expectations of the course

Almost all (*n* = 159; 97.5%) of the participants reported that the course met their expectations, whereas 2.5% (*n* = 4) reported that the course did not meet their expectations or had mixed feelings (Table 2).

**TABLE 2:** Participants’ expectations of the course.

Category	Code	*n*	Quote
Course exceeded participants’ expectations	Experiential and practical	48	‘Yes, the course exceeded my expectations! I expected to be taught a bit and have practical sessions but I did not expect such collaboration between lectures and practicals which really emphasized the concepts’. (Participant 22)
	Interprofessional experience	37	‘Yes, this course exceeded my expectations, since it wasn’t only focused on OT – treatment; I now feel more confident in areas that are more relevant to other professions (e.g. speech therapy) and will be able to treat these aspects since speech therapists are not available in our area’. (Participant 110)
Course met participants’ expectations	Applicable and appropriate	41	‘Yes, the course was very informative and covered a wide range of topics that was applicable to my profession as an occupational therapist. I actually learned about so much more that what I was thought I would’. (Participant 56)
	Informative and comprehensive	34	‘Yes, it has met my expectations because I have learnt everything I thought I need to know when working with CP children. I learned much more’. (Participant 7)
	Well-structured and organised	14	‘Yes. I found the course to be very well organised and run efficiently with very knowledgeable experienced therapists running it. The structure of the course is also impressive. I love the fact that the care givers are being trained on the same information. Brilliant!’ (Participant 58)
Mixed feelings	Practice time not adequate	1	‘In a way yes, in a way no. I came with the hope that I would be able to more effectively treat my CP kiddies. (Which in essence I have learnt a lot), but in the context of my hospital and clinics it hasn’t helped as much as I thought it would. Ideally, I would love to be able to spend 30 min preparing the child’s body but that would practically take too long’. (Participant 11)
Course did not meet participants’ expectations	Less relevant to speech therapy	1	‘No. I thought the course was more physio[therapy] and OT oriented. As a speech therapist I mostly did not feel I played a big role or had my expertise maximized; topics covered were mostly related to physio[therapy] and OT’. (Participant 14)
	Speech therapy topics not covered adequately	2	‘No. I came on the course looking for more information on communication intervention. I thought this would be covered. The information covered in the communication lecture was not sufficient as it was everything that I already knew. I paid a lot of money to come on the course which was aimed on OTs/PTs only’. (Participant 70)

*n*, denotes the number of quotes associated with the code. OT, occupational therapy; PTs, physiotherapy or physiotherapists.

### Change in self-perceived level of knowledge regarding cerebral palsy

Following the course, there was a significant change in participants’ self-perceived level of knowledge (*p* < 0.001) in all five areas of CP covered in the course (Table 3).

**TABLE 3:** Participants’ self-perceived change in level of knowledge about cerebral palsy.

Category	Number	Score before course		Score after course		Score difference		*p*
		Mean	s.d	Mean	s.d	Mean	s.d	
Assessment	130	9.33	4.28	23.98	2.99	14.65	4.71	0.00
Treatment	130	5.55	2.88	16.16	2.12	10.61	3.28	0.00
Play and communication	131	17.58	9.59	41.92	4.79	24.32	8.81	0.00
Management	129	9.19	4.41	22.94	3.19	13.75	4.59	0.00
Cerebral visual impairment	131	3.95	3.40	15.69	2.39	11.75	3.79	0.00

s.d., standard deviation.

### Self-perceived change in attitude, perception and clinical practice intention

The analysis of qualitative data under the theme of perceived areas of change following the course revealed three categories. These were change in attitude, change in perception and change in clinical practice intentions. In the categories of change in attitude and perception, the participants expressed a willingness to adopt more positive attitudes and perceptions towards working with children with CP in terms of being more confident and positive in their reception and expectations of the children (Table 4).

**TABLE 4:** Participants’ self-perceived change in attitude and perception towards children with cerebral palsy.

Category	Code	Quote	
		Before I used to…	Now I will…
**Attitude**	Confidence in CP management	‘Feel overwhelmed and incompetent as soon as a child with CP came. I used to doubt myself and felt was not doing enough’. (Participant 2)	‘Be confident with CP and just remember that a child with CP is just a child’. (Participant 2)
		‘Worry about seeing a child with CP and feel nervous and unconfident when treating them’. (Participant 113)	‘Be calm and collected and have purposeful treatment using the principles of handling that I have learnt in this course!’. (Participant 113)
	Positive reception of children with CP	‘Be scared of children with CP and therefore cringe when they were referred. I always felt like I should know more but I didn’t’. (Participant 38)	‘Enthusiastically wait to discover what the child wants me to learn about him or her and how I can help them’. (Participant 38)
		‘Go into panic mode every time I saw a child with CP. Assessment and treatment were nightmares for me’. (Participant 42)	‘Feel excited when a CP kid arrives for therapy …confidently greet and smile at the child and caregiver’. (Participant 42)
	Empathy for caregiver	‘Jump to conclusions about what the caregivers were doing right/wrong and tell them what to do instead of looking at the whole picture and understanding why the caregiver does things the way that she does’. (Participant 93)	‘Use knowledge, the mom’s experience and the child’s potential to achieve functional, realistic goals … and also put myself in the shoes of those who have to care for this child and take into account the immense challenges they face. With understanding I hope to be a better therapist’. (Participant 93)
		‘Judge moms and carers, even previous therapists for not doing things right without caring’. (Participant 60)	‘Be patient and supportive to parents and listen to them more’. (Participant 60)
**Perception**	Seeing possibilities in a child	‘…[*N*]ot see the real potential of children with CP and not even enjoy the treating [*of*] the children’. (Participant 77)	‘…[*T*]hink of CP as a way of life, in which the child has a lot of possibilities with each passing day…and place expectations on the child’. (Participant 77)
		‘Simply do passive treatments, underestimate potential, do poor groups, teach irrelevant stuff and never touch Cerebral Visual Impairment’. (Participant 31)	‘Look forward to treat CP, talk a lot more with the children and give them time to respond. Now I know there are endless possibilities’. (Participant 31)
	Understanding the child’s needs	‘Start to treat the child before knowing exactly all the problems and difficulties the child has, positioning of children wasn’t effective and passive stretching’. (Participant 37)	‘Assess and observe the child before treating, position [*the*] child in the correct way for that specific classification. And also not do passives’. (Participant 37)
		‘Box all children with CP and think that all children with CP have somewhat cognitive involvement and would not have a good choice making concept’. (Participant 13)	‘…[*A*]dapt treatment to the child and offer more choice making and opportunities for the child to communicate’. (Participant 13)

CP, cerebral palsy.

In the category of change in clinical practice intentions, the participants reported several areas where they intended to change their way of practice. These areas could be further grouped into four subcategories: approach to child management, caregiver involvement, service delivery and continuity of care (Table 5).

**TABLE 5:** Participants’ self-perceived change in clinical practice intention regarding cerebral palsy management.

Subcategory	Code	Quote	
		Before I used to…	Now I will…
**Approach to child management**	Thorough assessment and classification	‘Speed through the assessment and not spend time observing the child before getting hands on’. (Participant 6)	‘Slow down [*and*] thoroughly assess each child and determine their classification’. (Participant 6)
		‘Just quickly assess the child and not classify them accordingly and mostly not classify them at all’. (Participant 119)	‘Use the classification systems to help understand my child and plan treatment better and also assess the child more thoroughly and note associated impairments’. (Participant 119)
	Holistic approach	‘Rush assessment and only concentrate on physio issues more, talk to parent/carers as if I always knew more based on training’. (Participant 102)	‘Assess my patient in a more holistic and organised manner with a more methodical guideline and communicate with the speech therapist and occupational therapist more’. (Participant 102)
		‘Have a speech therapy approach, using treatment techniques that fall in my scope of practice and mainly manage CP at impairment level’. (Participant 97)	‘Look at all aspects that affect the child’s life and treat them in a holistic manner. I will work in an MDT and communicate more with the OT and PT’. (Participant 97)
	Individualised therapy	‘To think that all children with CP have somewhat cognitive involvement and would not have a good choice making concept’. (Participant 22)	‘Adapt treatment to the child and offer more choice making and opportunities for the child to communicate’. (Participant 22)
		‘Box all children with CP and give the same advice to all moms’. (Participant 66)	‘Use knowledge, the mom’s experience and the child’s potential to achieve functional, realistic goals. Watch, wait and wonder in order to really see what a child can do’. (Participant 66)
	Goal directedness	‘I did not know how to comprehensively assess a child with CP in order to be able to determine priority treatment goals’. (Participant 84)	‘Use knowledge, the mom’s experience and the child’s potential to achieve functional, realistic goals’. (Participant 84)
		‘Have difficulty setting appropriate goals. I had no direction in treatment and certainly did not consider the fact that CP was a way of life’. (Participant 24)	‘Thoroughly assess each child and determine their classification. Based on that, determine sufficiently handling principles and set up goals with the parents. Plan treatment so that it can become a way of life and be continued every day at home for the rest of the lives, thus focus on caregiver involvement’. (Participant 24)
	Child engagement	‘…[*N*]ot to focus on the child’s ability, never involved the caregiver, and did not include play as a session (therapy goal)’. (Participant 16)	‘I will give the child opportunity…and allow the CP children to play and engage with the environment (increase play)’. (Participant 16)
		‘Only speak to the mom and rarely the child. I used to think that all children with CP have somewhat cognitive involvement and would not have a good choice making concept’. (Participant 20)	‘Adapt treatment to the child and offer more choice making and opportunities for the child to communicate’. (Participant 20)
	Creative use and making of assistive devices	‘Have no idea what to suggest to moms for them to use home as equipment as they are in such poor settings’. (Participant 14)	‘…[*G*]ive moms ideas and help with making inexpensive, effective and accessible toys and equipment’. (Participant 14)
		‘…[*J*]ust think there are no toys and resources and equipment’. (Participant 20)	‘I will definitely recommend it to my friends and make my own resources based on the information attained’. (Participant 20)
**Caregiver involvement**	Empowerment through education	‘Underestimate what children with CP can do and not always focus enough on parent education’. (Participant 86)	‘Treat CP as a way of life – ADL!! … let the caregiver interact with the child and run groups and so educate and empower caregiver’. (Participant 86)
		‘Not want to recommend anything to a Mom since I felt they handled their child best’. (Participant 45)	‘Really see potential in these kiddies, make parent education a large part of my treatment program’. (Participant 45)
	Collaboration with the mother	‘Feel hopeless and useless in my attempts to help mothers and could not assist in feeding’. (Participant 66)	‘Involve the mothers and carers more, ask their advice and include them more in decision-making so as to be able to handle CP in the best way possible, using the parent as the biggest team member in therapy’. (Participant 66)
		‘Never involve the moms and not train them better to understand their child and manage well at home’. (Participant 118)	‘Organise groups and attempt carer to carer training for continuation of groups in areas where we struggle to reach’. (Participant 118)
**Service delivery practice**	Improve record-keeping	‘Not have a good guideline for treatment and assessment. I would just use drips and drabs of information and apply it randomly’. (Participant 6)	‘Try standardise our CP assessment forms and work out an efficient CP database so [*that*] children are not neglected’. (Participant 6)
	Efficient use of available resources	‘Have no idea what to suggest to moms for them to use home as equipment as they are in such poor settings’. (Participant 39)	‘…[*G*]ive moms ideas and help with making inexpensive, effective and accessible toys and equipment’. (Participant 39)
		‘Just think there are no toys and resources and equipment’. (Participant 20)	‘Do better group therapies to maximise use of the little time and equipment available’. (Participant 20)
**Continuity of care**	Home rehabilitation	‘Forget about the future and focus on a thing that cannot be necessarily changed’. (Participant 82)	‘Look more holistically, include caregiver much more and empower them. I will think about the future and 24-hour management’. (Participant 82)
		‘Briefly assess highest functional level and find missing component, treat the impairment like a robot without thinking about handling, future, communication or effectiveness of what I was doing in relation to reality for that patient, i.e. only coming to therapy once a month’. (Participant 91)	‘Organise groups and attempt carer to carer training for continuation of groups in areas where we struggle to reach’. (Participant 91)
	Social environment to inform intervention	‘… [*N*]ot know how to use the home environment for CP children’. (Participant 68)	‘…[*E*]xplore possibilities for going on home visits to observe and assess child in the natural environment’. (Participant 68)

CP, cerebral palsy; OT, occupational therapist; ADL, activities of daily living; MDT, multi-disciplinary team; PT, physiotherapy.

### Elements identified by participants as essential components of a short practical course

Four subcategories were identified under the category of ‘aspects of the course that participants considered essential’. These included adult teaching and learning methods, conducive learning environment, holistic approach and relevant course curriculum (Table 6).

**TABLE 6:** Elements identified by participants as essential components of the course.

Subcategory	Code	Quote
**Adult teaching and learning methods**	Practical application of theory	‘The structure of the course – being able to hear the theory, see photos of case examples, work with a mother and child and then discuss therapy sessions – helped me think through problems. CP as a way of life really brought everything together for me’. (Participant 18)
		‘Getting hands-on experience working with a child and CP and to start developing handling techniques’. (Participant 11)
	Working with an actual child	‘The large amount of clinical practicals and continuous involvement with the same child really allowed me to learn better’. (Participant 106)
		‘Having hands-on time with actual children with CP and building relationships with the child and mother really helped me a lot’. (Participant 92)
	Involvement of a caregiver	‘I loved having a variety of aspects covered during the course, the videos, and having the caregivers and mothers with us during the sessions’. (Participant 80)
		‘I really enjoyed the fact that the mothers/caregivers were also trained at the same time and thus did not just receive information from us but from a trained caregiver’. (Participant 23)
	Constant feedback	‘Practical experience; well-organised theory notes for reference, lots of feedback from all participants and experience with a variety of different children, diagnoses and levels of functioning stood out for me’. (Participant 61)
		‘The group discussions and feedback on everything, the individual practical groups on each topic covered, the supervision and freedom to ask questions made the course a great experience’. (Participant 87)
	Demonstrations by experts	‘All the standardised classification tools, the hip surveillance information and hands-on practical demonstrations; so helpful to watch and learn from skilled specialised therapists’. (Participant 54)
		‘Practical experience/sessions and handling. Theory on subtypes, hands-on approach of supervisors when helping during the prac sessions. I learned a lot being able to watch how Gillian/Lindy handled the child’. (Participant 48)
	Sharing amongst peers	‘The process of working with a child throughout the course as this was like a crash course in what the assessment and treatment planning phases in a CP child would likely involve. The sharing between participants allowed for exposure to a variety of CP children’. (Participant 33)
		‘Working with participants from other disciplines really allowed exchange of knowledge and skills, I appreciated more things that I never used to pay attention to’. (Participant 7)
**Teaching and learning environment**	Comfortable environment	‘The openness and freedom to engage with the Malamulele members and ask questions… It was also really helpful to have a facilitator and someone to ask questions. It was very nice how we didn’t feel any question was stupid’. (Participant 60)
		‘It was amazing to learn from the team and share ideas in a comfortable environment and get feedback and guidance’. (Participant 19)
	Presenters perceived as experts in the field	‘All the standardised classification tools, the hip surveillance information and hands-on practical demonstrations; so helpful to watch and learn from skilled specialised therapists’. (Participant 54)
		‘I loved the practice sessions. It was amazing to apply the theory and to also learn from the experts in the Malamulele therapy team! To be able to share ideas in a comfort environment and get feedback and guidance’. (Participant 11)
**Multidisciplinary approach and interprofessional learning**	Mixed therapists allowed peer learning and sharing	‘…I liked that we had mixed therapists to have range of ideas and skills, holistic approach and education. Theory, then practical, then feedback. It was amazing to learn from the team and share ideas’. (Participant 82)
		‘It was helpful to work with other disciplines, along with using the different methods of learning: pracs, videos, lectures, group discussions’. (Participant 72)
	The different disciplines gave holistic view	‘I found the holistic view of the course most useful. Instead of just learning about speech therapy-related assessment and treatment, I was exposed to positioning, fine and gross motor assessment and management relating to OTs and PTs which are still relevant, if I were handling a CP child of my own’. (Participant 12)
		‘…I liked that we had mixed therapists to have range of ideas and skills, holistic approach and education’. (Participant 4)
**Relevant curriculum content**	Classification of CP	‘I learned the importance of assessing and classifying a child with CP the correct way and also learned how to do this; it is great to now be able to use this to guide my treatment to ensure better functionality’. (Participant 3)
		‘I appreciated learning how to classify a child with CP, make equipment especially for your child, feeding and learning about the sensory stimulation box. Seeing all the different videos of the various classifications. All the practical demonstrations’. (Participant 27)
	Cerebral visual impairment	‘I did not have any practical exposure at my university in CP. Before the course I thought CVI stood for Cerebral Vascular Infarct. The course was informative and since it was rural based, it will make the skills that I have learnt very useful and applicable to us in the rural setting’. (Participant 44)
		‘I really liked gaining skills which I previously thought had to remain in specific disciplines, e.g., CVI, feeding and sensory stimulation’. (Participant 54)
	Preparation of the child	‘I found the loosening and preparation techniques useful as I didn’t know them before’. (Participant 71)
		‘I have learnt methods of preparation which will make it easier for me to cast for splints, also the importance of the bond between mother and child’. (Participant 111)
	The concept of looking at CP as a way of life	‘I found it eye-opening to realise that CP is a way of life and everyday activity is a part of treatment and management’. (Participant 12)
		‘Looking at CP as a way of life really brought everything together for me’. (Participant 31)
	Assistive devices and equipment	‘The practical techniques in feeding and different communication tools and the making of low-cost equipment and toys’. (Participant 9)
		‘Learning about classifications of CP… making assistive devices from cardboard Wow! The practicals on devices were useful’. (Participant 66)
	Postural management	‘I found the information on postural management and handling most useful as our skills at university did not cover this well but it was good to recap on everything’. (Participant 11)
		‘I loved postural management and handling of the children. It changed my view on the condition’. (Participant 82)
	Communication and feeding	‘All the topics that were covered were very useful to me. But especially the communication, feeding, posture and vision. I could relate to them easily’. (Participant 67)
		‘I loved everything, mostly the fact that we were taught how to communicate with children and to be able to classify them accordingly’. (Participant 8)
	Physical handling	‘… [P]hysical handling of CP children, warm up, how to take therapy to a home environment and incorporating CP as a “way of life,” stretching, massage, learning how to do trunk and limbs, observing Kabi’s session on play and learning about seating and how to use objects like buckets and basins to position children’. (Participant 41)
		‘Lectures on diagnosing CP and the subtypes, the principles of handling which is the first and foremost treatment and efficient intervention. Realising the uniqueness of every child and not to be too quick in putting the child in a clinical box’. (Participant 21)

CVI, cerebral vascular infarct; CP, cerebral palsy; PT, physiotherapy or physiotherapist OT, occupational therapy; prac, practical.

## Discussion

The profile of therapists attending this short course represents graduates from all South African training institutions who worked in eight of the nine provinces at the time of doing the course. Although not statistically representative of the larger population of newly qualified therapists in South Africa, the variation in the sample may have provided important insights especially in the qualitative data. More than two-thirds of the participants were therapists with less than 2 years of work experience. This demonstrates that newly qualified therapists were particularly interested in developing their skills through attending the short course. In agreement with this, studies have shown that CPD is especially important during the critical years when a professional transitions from being a student to being a practitioner (Singh et al. 2015). Newly qualified therapists particularly seek to develop confidence, professional identity, interpersonal skills and practical field-specific skills (Moores & Fitzgerald 2017). In South Africa, most therapists with less than 2 years of experience will be doing community service in rural or underserved areas where working conditions are relatively challenging (Naidoo et al. 2017; Singh et al. 2015). Hence, newly qualified therapists will be more attracted to CPD activities that offer them opportunities to learn through practical and interprofessional approaches (Naidoo et al. 2017), and this was confirmed in this study. Another factor accounting for this observation is that the Malamulele Onward practical training course was particularly targeted at newly qualified therapists and therapists with little experience in working with children with CP; hence, the fact that the majority of course participants had less than 2 years of experience is not that surprising and suggests that the organisation is reaching its target market.

A majority of the course participants were satisfied with the course, with some even expressing that their expectations had been exceeded. They felt that the course had been informative, comprehensive, experiential and practical, and a positive learning experience, with good learning resources. The interprofessional approach and close integration of theory and practice were qualities that exceeded the participants’ expectations. However, there were a few who were not satisfied with the course. They expressed that the course was more oriented to occupational therapy and physiotherapy, which suggests that they were speech therapists. They felt that the aspect of communication had not been addressed as much as they had expected. Whilst this may indeed be an area of training need for speech therapists, the interprofessional nature of the course may not have allowed for in-depth teaching of specific intra-professional topics. The aim of the course was to expose therapists to the roles of the other professions in a way that could reinforce team and holistic approaches. This specific finding may also suggest a need for profession-specific courses for speech therapists to adequately address this very specific technical skill. Nevertheless, there is room for improving the course in a way that could increase the intra-professional gains for speech therapists.

The initial self-perceived knowledge levels, attitudes, perceptions and clinical practice tendencies of participants in this study confirm that the newly qualified therapists in South Africa feel unprepared to work with complex conditions in low-resource settings and hence support the need for CPD as suggested by Naidoo et al. (2017) and Singh et al. (2015) in their studies concerning preparedness of newly qualified therapists. Moreover, just as previous studies have suggested gaps in the community and public health curricula of therapists in South Africa (Naidoo et al. 2017; Mostert-Wentzel et al. 2009a), this finding may also suggest gaps in undergraduate curricula in the area of CP.

The before-and-after evaluation showed a positive change in knowledge, attitude and clinical practice intention of the participants. These outcomes from short practical training courses are similar to those observed in a practical CPD course that targeted clinical decision-making and use of evidence-based practice in paediatric rehabilitation by physiotherapists in Rwanda (Clark et al. 2018), short skills training course for critical care for Sri Lankan physiotherapists (Tunpattu et al. 2018) and a practical course on evidence-based practice for Irish occupational therapists (Brangan, Quinn & Spirtus 2015). The before-and-after course assessments in these studies demonstrated improvement in knowledge levels, positive change in attitude and readiness to change clinical practice. This confirms that a targeted short practical training course is effective for positive change in a particular area.

In particular, the area of communication and play showed the greatest significant difference in self-perceived improved knowledge. This is not surprising as these elements were the competencies therapists were not confident with before the course. This was mostly attributed to perceived inability to interact with a child with a disability. This is similar to findings in a case report by Clark et al. (2018) describing an education intervention model for physiotherapists working in paediatric rehabilitation in Rwanda. During the education intervention, the concept of playing and communicating with the child during therapy seemed to be a ‘paradigm shift’ for Rwandan physiotherapists. Before gaining the practical knowledge regarding play and communication, they were observed to approach the treatment of a child in a ‘perfunctory and serious manner’ (Clark et al. 2018:7). This suggests that therapists have a poor background in training on the concepts of play and communication, particularly in children with a complex disability such as CP. Undergraduate training is probably limited in terms of allowing for adequate development of these specific skills. Whilst there is room for improving this side of undergraduate training, CPD platforms such as short practical training courses could also help bridge these gaps in such specialised areas as shown in this study.

It is likely that the four aspects of the course that participants identified as essential components contributed to their successful learning. They perceived that relevant course content was delivered using adult teaching and learning methods, including the large practical component, both peer and expert learning, and constant feedback and reflection. This is consistent with the CPD preference that South African allied health professionals have reported in a comparative study conducted by Van Vuuren and Nel (2013). In this study, therapists reported that they preferred CPD in the form of ‘small work groups rather than large formal lectures’ where they can engage in active learning and skills acquisition in relevant subjects (Van Vuuren & Nel 2013:45). The findings are also in agreement with the theories of adult learning described in the literature that hold the view that adults learn through practical engagement, peer sharing and expert feedback (Clark et al. 2018; Grace et al. 2017). Therefore, when designing short courses, the relevance of course content and principles of adult learning should be considered in order to meet the participants’ needs.

Participants in this study reported that the learning environment was conducive in that they felt comfortable and unthreatened in expressing their own ideas and making mistakes as they learned. They also felt that there was constant feedback, lots of sharing amongst peers and guidance from the facilitators whom they perceived as experts. This is consistent with adult learning theories which propose that adults prefer a learning environment that is safe, robust and accommodative (Gooding, Mann & Armstrong 2017; Grace et al. 2017). Therefore, as much as facilitators need to employ good and multiple teaching methods, they also need to ensure that the learning environment is conducive for learning.

The course was interprofessional with the unique feature of involving the caregiver as part of the team throughout the course. In this way, there was interaction and practical input from all members of the rehabilitation team participating in the course. This highlights the importance of interprofessional learning in fostering practical understanding of holistic healthcare. Interprofessional learning has been identified as a means of teaching undergraduate students to start learning how to collaborate and value shared decision-making and contribute in holistic patient care (Maharajan et al. 2017; Walkenhorst et al. 2015). This study has demonstrated how useful interprofessional learning can be even for qualified health professionals.

Moreover, the special feature of involving the caregiver resonates well with the FCA, which is the gold standard of child rehabilitation (Chiarello et al. 2016). The approach describes the mother (or child’s family) as having a crucial role in decision-making and implementation of rehabilitation plans (Chiarello et al. 2016; McDowell et al. 2015). Therefore, the relationship between therapists and the child’s family (caregiver) has been best described as a ‘partnership’, where there should be close collaboration throughout all processes and stages (Novak 2014). This has been demonstrated in this study, with participants recognising the caregiver as a source of ideas and insight into the child’s needs. Therefore, the interprofessional approach is an essential component to consider for managing and understanding such complex conditions as CP.

## Conclusion

Newly qualified therapists in this study perceived that a 6-day practical training course increased their level of knowledge, awareness of basic skills and confidence with CP management. They also perceived a positive change in their attitudes towards children with CP and their clinical behavioural intentions regarding CP management. Participants in this study also identified components of the short course that they found essential to their learning, namely, utilisation of adult teaching and learning principles such as experiential learning with an actual child with CP, provision of relevant course content such as content on communication and play, provision of a conducive learning environment and the holistic approach that was achieved through interprofessional learning.

### Implication of the findings

The findings of a lack of readiness amongst newly qualified therapists to manage CP shown in this study may be true for most newly qualified therapists in South Africa. In this study, most of them reported a lack of knowledge and skills transferable to settings with low resources, including the use of active (as opposed to passive) therapy modalities, equipment making and creativity in play and communication therapies. This could imply a need to strengthen undergraduate training inputs in these areas using methods such as experiential learning, interprofessional learning and mentoring. This study highlights CPD support in terms of short training courses on specific complex conditions like CP as a vital tool to be employed for all newly qualified therapists for the specific purpose of bridging the readiness gap during the transition from student to therapist. It is possible that the course would benefit any other long-serving therapists who may not have the necessary background for managing CP. However, further follow-up and mentoring are necessary to ensure the translation of new skills into routine practice.

### Limitations of the study

Owing to the nature of the study, member checking and test–retest reliability checks of the data collection tools were not done, which might have potentially limited the trustworthiness of the information gathered. Secondly, the way qualitative data were collected did not allow the researchers to draw comparisons between the three professions. Isolating profession-specific information would have been valuable for providing profession-specific feedback. In addition, all outcomes were self-perceived measures and not objective measures. This limited the insight into the actual effect of the course on the therapists. Moreover, although the study set out to focus on newly qualified therapists, it included records of therapists with up to 4 years of working experience, which may be considered significantly more than the experience of a community service therapist in South Africa. This may have accounted for significant differences in the outcomes of the study. Lastly, the study only involved a before-and-after training assessment, which provided no information as to what extent the course helped to change practice.

### Recommendations

Future research should be conducted to determine if gains from the course translate to actual change in clinical practice of the participants and improvements in actual patient outcomes. Future research should involve, for example, sending a 6-month follow-up questionnaire, or doing hospital visits to determine the extent to which participants retain and implement the gains from the course. Moreover, additional robust means of collecting data, such as focus groups, should be employed to get more in-depth and richer information on the value of the short practical courses in CP. In addition, comparative studies in this area should be structured in a way that clearly highlights how prior exposure to CP, differences in discipline of therapists and years of experience in CP management affect the study outcomes in a similar study.

Public health policies should promote CPD activities in complex areas of rehabilitation for newly qualified therapists. This could be in the form of provincial rehabilitation managers sponsoring therapists to attend such courses, hence improving the affordability and accessibility of such opportunities to newly qualified therapists.

## Appendix 2: Questionnaire on knowledge

**My Knowledge**

**How would you rate your knowledge in the following areas?**

**Name:**

**Table:**

Topic	After the training course
Understanding what CP is	1 2 3 4 5 6 7 8 9 10
How to assess a child with CP	1 2 3 4 5 6 7 8 9 10
How to set goals in treatment	1 2 3 4 5 6 7 8 9 10
How to classify a child with CP	1 2 3 4 5 6 7 8 9 10
How to treat a child who is stiff	1 2 3 4 5 6 7 8 9 10
How to treat a child who moves too much (dyskinetic)	1 2 3 4 5 6 7 8 9 10
How to make equipment for child with CP	1 2 3 4 5 6 7 8 9 10
How to play with a child with CP	1 2 3 4 5 6 7 8 9 10
Knowing what kind of toys are best for a child with CP	1 2 3 4 5 6 7 8 9 10
Understanding parents’ feelings	1 2 3 4 5 6 7 8 9 10
How to design a home programme	1 2 3 4 5 6 7 8 9 10
What to do on a home visit	1 2 3 4 5 6 7 8 9 10
Knowing how to recognise that a child has CVI	1 2 3 4 5 6 7 8 9 10
Ideas for how I can help a child with CVI	1 2 3 4 5 6 7 8 9 10
Understanding what is communication	1 2 3 4 5 6 7 8 9 10
Understanding the different ways children with CP may communicate	1 2 3 4 5 6 7 8 9 10
Ideas to help children with to understand and talk better	1 2 3 4 5 6 7 8 9 10
	

CP, cerebral palsy; CVI, cerebral vascular infarct.
